# Assessing toxicity and competitive fitness of *Vibrio* isolates from coastal waters in Israel

**DOI:** 10.1128/msphere.00025-25

**Published:** 2025-04-02

**Authors:** Katarzyna Kanarek, Kinga Keppel, Hadar Cohen, Chaya Mushka Fridman, Motti Gerlic, Dor Salomon

**Affiliations:** 1 Department of Clinical Microbiology and Immunology, School of Medicine, Faculty of Medical and Health Sciences, Tel Aviv University 616193, Tel Aviv-Yafo, Israel; Vanderbilt University Medical Center, Nashville, Tennessee, USA

**Keywords:** *Vibrio*, type VI secretion system, type III secretion system, MARTX, GMT island, antibacterial, virulence, toxin, antibiotic resistance

## Abstract

Several species of aquatic bacteria belonging to the genus *Vibrio* are emerging pathogens of humans and marine animals. *Vibrio*-associated infections have been shown to correlate with the increase in the oceans’ surface water temperatures. A slow yet steady increase in *Vibrio* isolates from clinical settings in Israel over the past decade led us to investigate their pathogenic potential in Israel’s coastal waters. We sequenced the genomes of 23 *Vibrio* isolates from the Mediterranean and Red Seas. Analysis of these genomes revealed the presence of diverse toxin secretion systems and toxins, as well as mobile genetic elements known to facilitate the dissemination of fitness-enhancing determinants. Moreover, we showed that at least 10 of these isolates induce cell death in bone marrow-derived macrophages and that at least 12 isolates intoxicate a rival *Vibrio* strain in interbacterial competition. Lastly, we determined the susceptibility profiles of these isolates to common antibiotics used to treat *Vibrio* infections and found widespread resistance to azithromycin. Taken together, our results reveal pathogenic potential within the *Vibrio* population of Israel’s coastal waters and underline the need for continued environmental monitoring of emerging pathogens.

**IMPORTANCE:**

The ocean’s surface water temperatures have increased in the past decades due to climate change. This increase correlates with the spread of *Vibrio*, a genus of aquatic bacteria, many of which are pathogens of humans and marine animals. Since *Vibrio*-associated illnesses are rising in Israel, we set out to investigate the *Vibrio* population in Israel’s coastal environments and monitor their pathogenic potential. We found diverse repertoires of predicted toxins, the ability to kill mammalian immune cells, and traits that enhance bacterial fitness, such as antibacterial toxicity and resistance to antibiotics commonly used to treat *Vibrio* infections. These findings indicate that pathogenic traits are circulating within the environmental *Vibrio* population in Israel’s coastal waters and suggest that continued monitoring is essential to identify emerging pathogenic strains.

## OBSERVATION

*Vibrio*, a genus of aquatic Gram-negative bacteria that inhabit marine and estuarine environments, includes several species pathogenic to humans and marine animals (e.g., *V. cholerae*, *V. vulnificus*, *V. parahaemolyticus*, and *V. palginolyticus*) (1). *Vibrio*-associated human infections, termed vibriosis, manifest as gastroenteritis, wound infections, or ear infections resulting from the consumption or handling of raw seafood and from recreational swimming (1). Notably, infections are prevalent during warmer months when *Vibrio* species thrive in the environment (2). Recent reports have highlighted a correlation between climate change-associated elevated sea surface water temperatures, the spread of pathogenic *Vibrio* species, and an increase in *Vibrio*-associated disease occurrence (2–4).

Annual reports from the Central Laboratories in the Israel Ministry of Health, which are tasked with microbial pathogen surveillance, reveal a slow yet steady increase in the number of *Vibrio* isolates from clinical settings in Israel over the last decade, primarily *V. cholerae* non-O1/O139 and *V. alginolyticus* isolated from ear and blood samples (5). Although research focusing on clinical and environmental *V. vulnificus* in Israel was conducted following a 1996 outbreak (6), the environmental *Vibrio* population in Israel’s coastal waters, a potential reservoir of pathogenic strains, remains understudied.

In the current study, we set out to investigate the toxic and competitive fitness potential of environmental *Vibrio* isolates in Israel. To this end, we collected coastal water samples from two sites in the Mediterranean Sea (Tel Aviv-Yafo and Ma’agan Michael) and one site in the Red Sea (Eilat) during the summer of 2023 and recovered *Vibrio* colonies growing on selective TCBS media plates (Fig. S1; Supplementary Methods). Of these, 23 colonies with diverse morphologies were selected for whole-genome sequencing (Table S1). Average nucleotide identity (ANI) analyses revealed that the isolates belong to various *Vibrio* species, including *V. alginolyticus*, *V. vulnificus*, *V. parahaemolyticus*, *V. campbellii*, *V. fortis*, *V. chagasii*, *V. owensii*, *V. mediterranii*, *V. diabolicus*, and *V. harveyi*. One isolate, *Vibrio* sp. TBV020, had an ANI score below 95% compared to the genomes available in the JSpeciesWS genome database, suggesting that it represents a new species (7). We named it *Vibrio sessaei* in honor of our late colleague, Prof. Guido Sessa (this name has not yet been officially approved).

Various virulence factors have been described in the *Vibrio* pan-genome, amongst them the type III secretion system (T3SS) (8), the type VI secretion system (T6SS) (9–11), and diverse arsenals of secreted toxins (e.g., MARTX toxins [12] and Cholera toxin [13]) (1). Notably, many of these virulence factors are encoded on mobile genetic elements (MGEs) that have been shown to be horizontally transferred between *Vibrio* species, thus posing a risk for the emergence of new pathogens (14).

**Fig 1:**
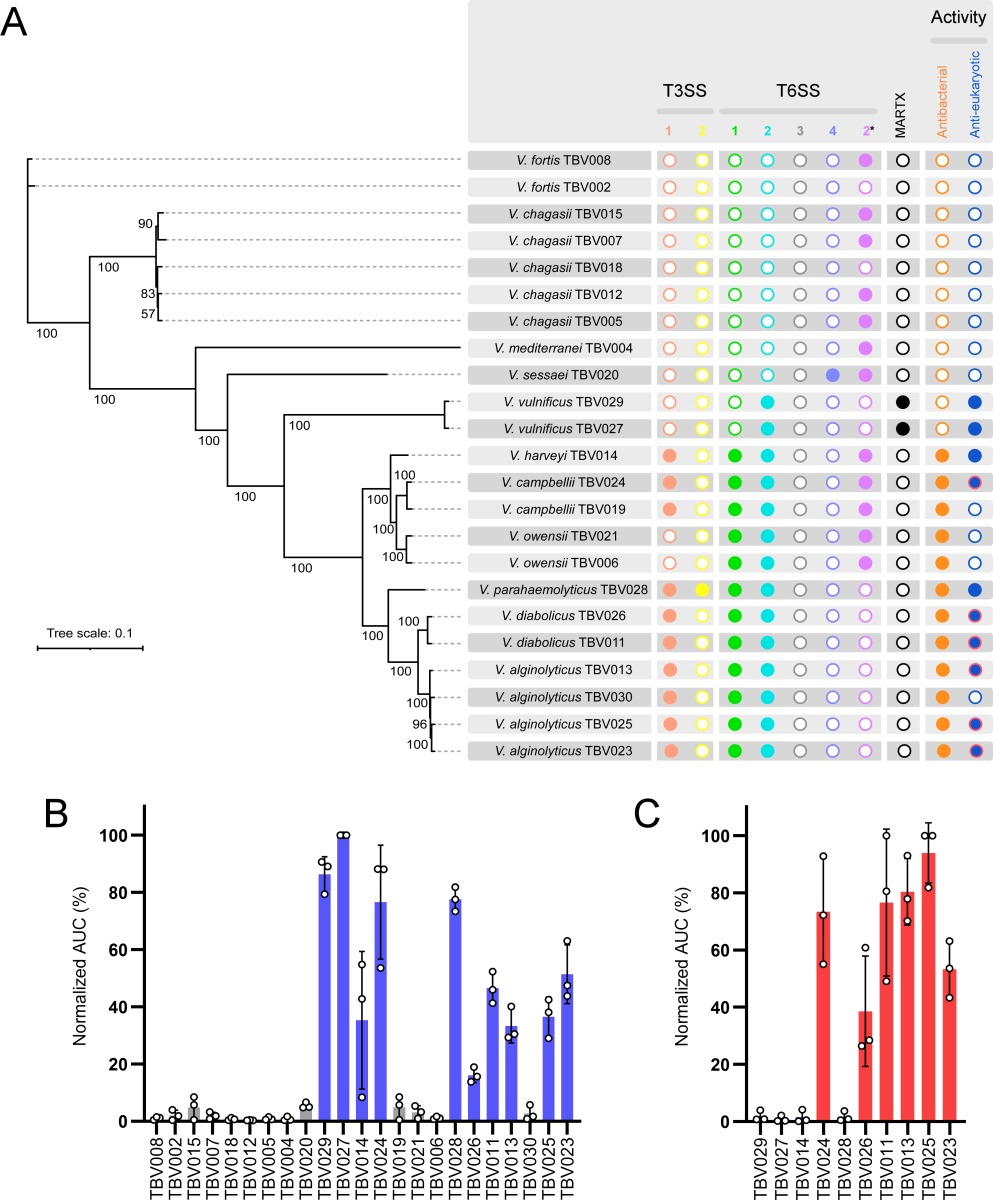
Environmental *Vibrio* isolates harbor toxin delivery systems and induce cell death in macrophages. (A) A summary of T3SSs, T6SSs, and MARTX toxins found in the genomes of the 23 indicated isolates. A full circle denotes the presence of the system or toxin. The ability of each isolate to induce cell death in macrophages (anti-eukaryotic; red circles denote contact-independent toxicity) or to intoxicate a competing *V. natriegens* bacterium (antibacterial) is shown on the right. T3SS1 and T3SS2 denote similarity to the two T3SSs known in *V. parahaemolytics*; in T6SS, numbers 1–4 denote similarity to T6SS1-4 from *V. parahaemolyticus*, whereas 2* denotes similarity to T6SS2 from *V. coralliilyticus*. The phylogenetic tree is based on the alignment of 2,296 core proteins found in the indicated isolates. The evolutionary history was inferred using the maximum likelihood method. Bootstrap values appear next to the corresponding branch as a percent of 100 replicates. (B and C) Assessment of cell death upon infection of bone marrow-derived macrophages (BMDMs). Approximately 3.5 × 10^4^ BMDMs were seeded into 96-well plates in triplicate and infected with the indicated *Vibrio* isolates (B) or the cleared media of overnight-grown isolates (C). Propidium iodide (PI) was added to the medium prior to infection, and its uptake kinetics were monitored for 5 hours using real-time microscopy (IncucyteZOOM). Cell death was determined as the area under the curve (AUC) of the percentage of PI-positive cells normalized to the number of cells in the wells. Results are shown as the mean ± SD of three independent experiments. An isolate was considered toxic with an average AUC >15%.

We analyzed the genome sequences of the 23 isolates for potential virulence factors, focusing on T3SSs, T6SSs, and MARTX toxins, each of which can simultaneously deliver a repertoire of toxic activities into host cells (Fig. 1A). Ten isolates, belonging to the species *V. alginolyticus*, *V. campbellii*, *V. harveyi*, *V. diabolicus*, and *V. parahaemolyticus*, harbor a T3SS similar to the well-studied T3SS1 from *V. parahaemolyticus* (15) (Fig. S2). The *V. parahaemolyticus* TBV028 isolate also harbors a T3SS2 (Fig. S3) previously shown to induce gastroenteritis (16, 17). In addition, the two *V. vulnificus* isolates harbor a MARTX toxin with toxic domains, such as C58-PaToxP-like (18) and RtxA-like/C2-2-like, which are predicted to intoxicate the host cells. Of the five T6SS types that have been characterized in *Vibrio* pan-genomes, we identified gene clusters representing four types (Fig. S4; Data set S1). Gene clusters comparable to *V. parahaemolyticus* T6SS1, T6SS2, and T6SS4 (19) are found in 12 isolates, 14 isolates, and one isolate, respectively (Fig. S5–S7). Clusters comparable to T6SS2 of *V. coralliilyticus* (9) are found in 12 isolates (Fig. S8). The latter was recently shown to target eukaryotes (9), whereas the *V. parahaemolyticus* T6SS2 and T6SS4 were predicted to target bacteria (19, 20). *V. parahaemolyticus* T6SS1-like systems were shown to deliver both anti-eukaryotic and antibacterial toxins (21, 22).

To investigate the toxic potential of the 23 isolates, we determined their ability to intoxicate murine bone marrow-derived macrophages (BMDMs), serving as model eukaryotic immune cells. We monitored cell death kinetics using real-time microscopy and found that 10 out of the 23 isolates were detrimental to BMDMs within 5 hours of infection (Fig. 1B; Fig. S9). The toxicity appears to be contact-independent for at least six of these isolates, as evidenced by cell death observed when BMDMs were incubated with cleared media in which bacterial cultures were grown overnight (Fig. 1C; Fig. S10). These results suggest that a secreted toxin is responsible for the toxic phenotype of these six isolates. Surprisingly, the presence of the anti-eukaryotic T3SS1 did not coincide with cell death, as demonstrated by isolates TBV019 and TBV030. Moreover, although the presence of a MARTX toxin in the two *vulnificus* isolates is predicted to induce contact-independent cell death, no effect was observed. Several factors can account for these discrepancies: (i) the growth or infection conditions used in our assays do not induce expression of the relevant systems; (ii) the systems are not functional; (iii) some isolates require longer than 5 hours to induce cell death; and (iv) BMDMs are not an appropriate target cell for some isolates.

**Fig 2:**
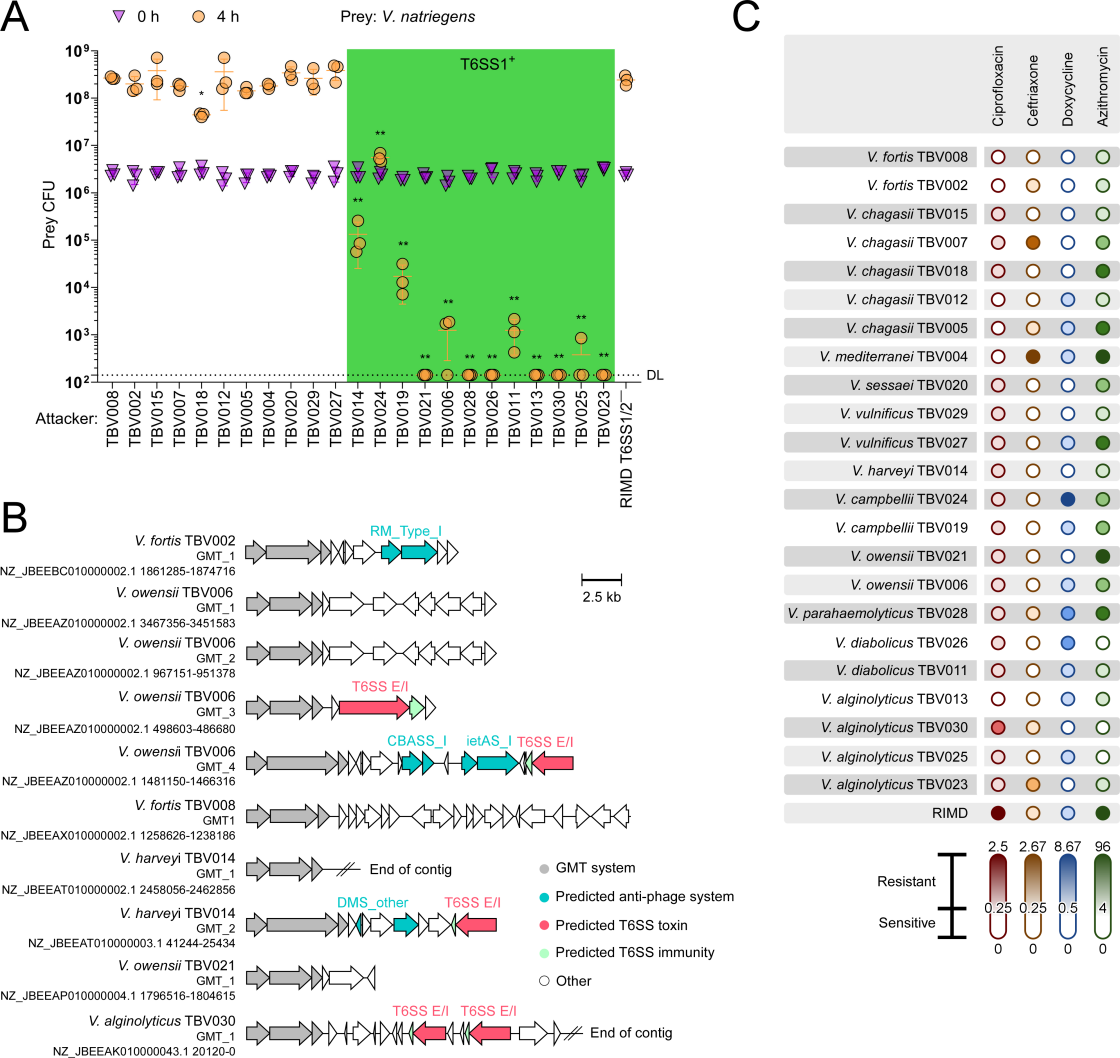
Antibacterial activity and antibiotic resistance in environmental *Vibrio* isolates. (A) Viability counts (colony-forming units [CFU]) of *V. natriegens* prey strains before (0 hour) and after (4 hours) co-incubation with the indicated *Vibrio* isolate attackers on MLB plates at 30°C. The statistical significance between samples at the 4-hour time point was calculated using one-way ANOVA with Dunnett multiple comparisons test on log-transformed data, compared to a *V. parahaemolyticus* RIMD 2210633 strain in which the two T6SSs were inactivated (RIMD T6SS1/2^−^). * *P* = 0.007; ** *P* < 0.0001. Data are shown as the mean ± SD of three biological samples; a representative experiment out of three independent experiments is shown. DL, the assay’s detection limit. (B) The gene structure and content of GMT islands found in the environmental isolates. RefSeq accessions are denoted. (C) The minimum inhibitory concentrations (MICs) of ciprofloxacin, ceftriaxone, doxycycline, and azithromycin were determined for each environmental isolate and the clinical *V. parahaemolyticus* RIMD 2210633 (RIMD) strain using ETEST strips. MIC values are displayed as color gradients to represent the resistance levels of each isolate. White color denotes susceptibility; increasingly darker shades denote resistance and higher MIC values. Sensitivity thresholds were set based on EUCAST guidelines, with the following ranges defining susceptibility: 0–0.25 µg/mL for ciprofloxacin and ceftriaxone, 0–0.5 µg/mL for doxycycline, and 0–4 µg/mL for azithromycin. The data shown are the mean of the three independent experiments.

Next, we sought to assess the competitive fitness of the isolates, since the ability to outcompete rival bacteria has been shown to indirectly contribute to virulence in other bacterial species (11). When co-incubated on solid agar plates, 13 isolates either killed or impaired the growth of another marine bacterium, *V. natriegens*, compared to a *V. parahaemolyticus* RIMD 2210633 strain in which the two antibacterial T6SSs were inactivated (Fig. 2A) (20). Notably, this antibacterial toxicity coincided with the presence of a T6SS1-like cluster (Fig. 1A), which was previously shown to be active in *Vibrio* species under the assay conditions (i.e., 3% [wt/vol] NaCl in the media, at 30°C) (9, 20). Furthermore, we recently described MGEs, known as GMT islands, that are prevalent in *Vibrio* species, where they serve as mobile armories containing antibacterial T6SS toxins and antiphage defense systems that enhance competitive fitness and protect against bacteriophage predation, respectively (23). Remarkably, we identified 10 GMT islands distributed across 6 of the 23 isolates (Fig. 2B). Isolate TBV006 harbors four GMT islands; although two of these islands are identical, they are located in different syntenic regions (Fig. S11). Analysis of the islands’ cargo revealed diverse antibacterial and antiphage arsenals, confirming our previous findings and indicating that these mobile armories can disseminate within local *Vibrio* populations to enhance competitive fitness.

Lastly, we assessed the susceptibility of the isolates to four antibiotics commonly used to treat vibriosis: ciprofloxacin, ceftriaxone, doxycycline, and azithromycin (Fig. 2C; Data set S2) (1). The results revealed diverse resistance patterns, with no single strain being sensitive or strongly resistant to all antibiotics, although over half of the isolates display strong resistance to at least one drug. Notably, resistance to azithromycin was particularly prevalent, as evidenced in 16 isolates.

### Conclusions

In this study, we isolated 23 *Vibrio* strains from Israel’s coastal waters and demonstrated that they wield toxic activities that may enable them to infect eukaryotic hosts. Moreover, we showed that many of the isolates possess antibacterial determinants, which may enhance their competitive fitness in the environment and indirectly contribute to their ability to colonize a host. Furthermore, we observed resistance to several clinically relevant antibiotics within this sample population. Taken together, our results demonstrate the pathogenic potential of the *Vibrio* population in Israel’s coastal waters and underline the need for continued surveillance of these habitats for the emergence of new pathogens.

## Data Availability

The genome sequences of the 23 *Vibrio* isolates described in this work were deposited to NCBI (Table S1) and are available with the following GenBank assembly accessions: GCA_046056525.1, GCA_046057245.1, GCA_046057235.1, GCA_046057115.1, GCA_046057095.1, GCA_046057045.1, GCA_046057055.1, GCA_046057035.1, GCA_046057005.1, GCA_046056995.1, GCA_046056975.1, GCA_046532705.1, GCA_046056945.1, GCA_046056895.1, GCA_046056935.1, GCA_046056605.1, GCA_046056745.1, GCA_046532695.1, GCA_046056635.1, GCA_046056645.1, GCA_046532685.1, GCA_046532655.1, and GCA_046056815.1.

## SUPPLEMENTAL MATERIAL

Data set S1 msphere.00025-25-s0001.xlsx TssB sequences used to generate a phylogenetic tree.

Data set S2 msphere.00025-25-s0002.xlsx Antibiotic resistance raw data.

Supplemental Information msphere.00025-25-s0003.pdf Supplemental methods, figures, tables, data set captions, and references.

ASM does not own the copyrights to Supplemental Material that may be linked to, or accessed through, an article. The authors have granted ASM a non-exclusive, world-wide license to publish the Supplemental Material files. Please contact the corresponding author directly for reuse.
